# The 7-α-dehydroxylation pathway: An integral component of gut bacterial bile acid metabolism and potential therapeutic target

**DOI:** 10.3389/fmicb.2022.1093420

**Published:** 2023-01-09

**Authors:** Journey L. Wise, Bethany P. Cummings

**Affiliations:** ^1^Department of Biomedical Sciences, Cornell University, Ithaca, NY, United States; ^2^Department of Surgery, Center for Alimentary and Metabolic Sciences, School of Medicine, University of California, Davis, Sacramento, CA, United States

**Keywords:** 7-α-dehydroxylation, bile acid, metabolism, microbiome, diabetes, bai operon

## Abstract

The gut microbiome plays a significant role in maintaining host metabolic health through the production of metabolites. Comprising one of the most abundant and diverse forms of gut metabolites, bile acids play a key role in blood glucose regulation, insulin sensitivity, obesity, and energy expenditure. A central pathway in gut bacterial bile acid metabolism is the production of secondary bile acids *via* 7-ɑ-dehydroxylation. Despite the important role of 7-ɑ-dehydroxylation in gut bacterial bile acid metabolism and the pathophysiology of metabolic disease, the regulation of this pathway is not completely understood. This review aims to outline our current understanding of 7-ɑ-dehydroxylation and to identify key knowledge gaps that will be integral in further characterizing gut bacterial bile acid metabolism as a potential therapeutic target for treating metabolic dysregulation.

## Introduction

### Overview

Projected to affect over 600 million adults worldwide by 2040, type 2 diabetes is a prevalent and rapidly growing chronic health condition associated with a multitude of serious co-morbidities (Goyal and Jialal, 2022). Accordingly, the widespread prevalence and life-threatening nature of type 2 diabetes necessitates the development of new and more effective strategies to address metabolic dysregulation. Characterized by inadequate production of insulin by pancreatic β-cells and a faulty response to insulin by insulin-sensitive tissues throughout the body, type 2 diabetes comprises nearly 90% of all diabetes cases. Though the pathophysiology of type 2 diabetes is likely multifaceted, research suggests that the gut microbiome plays a significant role in the maintenance of metabolic health, in large part through the metabolites that it produces. Bile acids are one of the most abundant and variable gut bacterial metabolites and have an increasingly appreciated role in the regulation of whole-body glucose homeostasis (Tomkin and Owens, 2016). However, despite their involvement in the pathophysiology of metabolic disease, our understanding of gut bacterial bile acid metabolism remains incomplete.

For background, primary bile acids are synthesized in the liver from cholesterol and, in humans, include cholic acid (CA) and chenodeoxycholic acid (CDCA). Before active secretion from the liver, primary bile acids are conjugated to taurine or glycine by amino acid *N*-acyltransferase. Following conjugation, bile acids are secreted by hepatocytes through the canicular membrane to be stored in the gallbladder for later secretion and digestion of lipids. Most bile acids (~95%) are then reabsorbed into the portal vein within the distal gastrointestinal tract and cycled back to the liver in a process known as enterohepatic recirculation. However, within the distal gastrointestinal tract, bile acids also interact with the gut microbiome, which performs a wide variety of metabolic modifications including the conversion of primary to secondary bile acids. These primary bile acids must first be deconjugated through the action of bile salt hydrolase (BSH) and are then converted into secondary bile acids, deoxycholic acid (DCA) and lithocholic acid (LCA), through a multi-step process known as 7-ɑ-dehydroxylation (Chiang, 2013). While the field has achieved a comprehensive, yet still evolving, understanding of gut bacterial bile acid deconjugation, our understanding of the more complex process of 7-ɑ-dehydroxylation remains incomplete. This review aims to outline our current knowledge of gut bacterial bile acid metabolism, focusing on the 7-ɑ-dehydroxylation pathway.

### Gut bacterial bile acid metabolism in metabolic health

The human gut microbiome is an incredibly complex environment containing between 10^13^ and 10^14^ bacterial cells and up to 1,000 different bacterial species (Kho and Lal, 2018; Eisenstein, 2020). Studies investigating the impact of the gut microbiome on metabolic disease have identified bile acids as key mechanistic mediators. For example, work in a mouse model of bariatric surgery has revealed that increases in cholic acid-7-sulfate produced by the gut microbiome contributes to the metabolic benefits of bariatric surgery (Chaudhari et al., 2021). Furthermore, dietary fiber supplementation has been reported to enhance gut bacterial 6-α-hydroxylation of bile acids to improve metabolic phenotypes in mice (Makki et al., 2022). Centenarians, individuals who exhibit decreased susceptibility to aging-associated illnesses, have also been shown to have a microbiome enriched for bacteria capable of producing various isoforms of lithocholic acid (LCA; Sato et al., 2021). In addition, a human clinical study suggests that fecal microbiota transplantation protects against the development of metabolic disease in patients with obesity which is associated with increases in gut bacterial bile acid metabolism (Allegretti et al., 2020, 2021).

Gut microbes perform numerous different modifications of luminal bile acids and regulate host metabolic health by determining downstream signaling, as bile acid subtypes vary in their affinity for bile acid receptors. The primary receptors involved in bile acid signaling include the nuclear receptor, Farnesoid X receptor (FXR), and the G protein-coupled receptor, TGR5, both of which are implicated in the regulation of glucose homeostasis and energy balance (Holter et al., 2020). Interestingly, the secondary bile acids, LCA and DCA, are the most potent natural agonists of TGR5 and thus represent promising bile acid metabolites for improving glucose regulation (González-Regueiro et al., 2017). For a more comprehensive overview of bile acid receptor signaling and the implication of these signaling pathways on metabolic homeostasis, the reader is referred to prior reviews on this topic (Claudel et al., 2005; Holter et al., 2020).

The biotransformation of primary bile acids to secondary bile acids by the gut microbiome occurs *via* a multi-step pathway involving deconjugation by bile salt hydrolase (BSH) followed by 7-ɑ-dehydroxylation. Termed the “gateway reaction” of gut bacterial bile acid metabolism, deconjugation of conjugated bile acids by BSH facilitates the formation of unconjugated bile acids that are capable of being modified *via* an array of metabolic pathways, including sulfation, dehydrogenation, and dehydroxylation (Jones et al., 2008). While bile salt hydrolases (BSHs) have been shown to deconjugate glycine and taurine-conjugated primary BAs, variation in the kinetics of this mechanism and BSH character has provided evidence to suggest a role for substrate specificity in the deconjugation of primary BAs (Ridlon et al., 2006; Guzior and Quinn, 2021). Bile salt hydrolases have been identified and isolated from a number of Gram-positive and Gram-negative bacterial species, including *Bacteroides*, *Clostridium*, *Lactobacillus*, *Bifidobacterium*, and *Listeria* (Ridlon et al., 2006). While all main bacterial phyla have been shown to include enzymes that catalyze deconjugation, evidence suggests that *Bacteroides* spp. are substantially involved in this reaction (Ovadia et al., 2019). The importance of the deconjugation step as a fundamental element of gut microbial bile acid metabolism is further supported by the omnipresence of genes encoding BSHs in gut bacterial species, as over a quarter of identified bacterial strains in the human gastrointestinal tract have been shown to facilitate BSH activity (Song et al., 2019). Further, metagenomic analyses by Jones et al. (2008) identified functional BSH in all major bacterial divisions and species of archaea present in the gut microbiome. Though variation in mechanism and composition of BSHs complicates our understanding of the specific impacts of BSHs on the gut microbiome, proposed theories suggest that deconjugation may serve a role in detoxifying bile salts, as well as optimizing colonization of the lower GI tract (Ridlon et al., 2006). Within the scope of this review, DCA and LCA are the most abundant secondary bile acids and function as substrates in subsequent 7-α-dehydroxylation (Hamilton et al., 2007; Ridlon et al., 2014).

While a limited number of bacterial strains capable of performing 7-α-dehydroxylation have been isolated and characterized, the complete array of bacterial species with 7-α-dehydroxylation functionality remains incompletely understood. Prior studies have identified enzymes involved in 7-α-dehydroxylation from *Lachnospiraceae* and *Peptostreptococcaceae* strains, and metagenomic analyses have found bai gene clusters primarily in *Firmicutes bacterium*, as well as *Lachnospiraceae* and *Peptostreptococcaceae* (Vital et al., 2019). An earlier study by Kitahara et al. (2000) suggested that *Eubacterium* may perform 7-α-dehydroxylation as well; however, the *Eubacterium* species that was assessed for 7-α-dehydroxylation activity was later re-classified as *Clostridium*. Nevertheless, recent literature suggests that there are likely bacterial strains that are capable of 7-α-dehydroxylation that are yet to be discovered (Vital et al., 2019). Interestingly, while the majority of currently available literature focuses on 7-α-dehydroxylation in *C. scindens*, a member of the *Peptostreptococcaceae* family, only a small percentage of bai gene-associated reads from recent metagenomic analyses were from this bacterial family (Vital et al., 2019). Our current understanding of 7-α-dehydroxylation suggests that the pathway is encoded by a bile-acid-induced (bai) operon that contains eight genes: *baiB*, *baiCD*, *baiE*, *baiA*, *baiF*, *baiG*, *baiH*, and *baiI*. Additional bai genes have been identified including *baiJ*, *baiK*, *baiL*, and *baiN* (Ridlon et al., 2006).

While this review is largely focused on the importance of gut bacterial bile acid metabolism in the context of metabolic health in humans, it is important to recognize that the relationship between bile acids and the microbiome is complex and that this complexity adds multiple additional layers of significance to our understanding of gut bacterial bile acid metabolism. For example, while gut bacterial bile acid metabolism influences the bile acid profile, there is also a bidirectional influence of bile acids on gut microbial profile as some bacteria are better able to survive in the presence of bile acids than others. The importance of this bidirectional relationship is further highlighted by research showing that gut bacterial bile acid metabolic potential is a key determinant of whether an FMT treatment will effectively colonize the host (Kakiyama et al., 2013; Ng et al., 2020).

Bile acids represent an attractive therapeutic target for the treatment and management of metabolic dysregulation as alterations in gut microbial composition have been associated with a number of disease states, including obesity, type 2 diabetes, polycystic ovarian syndrome (PCOS), and non-alcoholic fatty liver disease (NAFLD), among others (Wu et al., 2021). However, the development of agonists for bile acid receptors has been met with limited success due to the onset of negative side effects. Thus, the gut microbiome provides a means with which to target endogenous bile acid metabolism that is less likely to induce adverse effects. A variety of modalities have been proposed for the therapeutic modulation of bile acids, including both dietary and drug-based interventions, as well as genetic manipulation of gut bacteria (Guo et al., 2019; Wu et al., 2021). Furthermore, dietary changes induce compositional changes in gut microbiota and in circulating bile acid pools. One example among many, Pi et al. (2020) showed that a high-protein diet resulted in higher quantities of secondary bile acids *via* an increase in a 7-ɑ-dehydroxylating species of *Eubacterium*. Moreover, though the bidirectional relationship between gut-directed pharmaceutical therapies and gut microbial bile acid metabolism is less well-understood, conditions characterized by metabolic dysregulation are frequently treated with drugs that impact microbiota composition. As such, several studies have revealed associations between metformin use and gut microbial bile acid metabolism-related changes, including increased expression of BSH, glycoursodeoxycholic acid levels, and abundance of particular bacterial species (*Lactobacillus* and *Escherichia*) (Wu et al., 2017; Bauere et al., 2018; Sun et al., 2018). Furthermore, while genetic manipulation of bacterial species is limited in scope due to current knowledge gaps, several studies have successfully altered bile acid levels and metabolic capabilities of bacterial species *via* genetic modification (Guo et al., 2019; Funabashi et al., 2020). Despite the promise of gut microbial bile acid metabolism as a therapeutic target, limitations in our understanding of this process make it difficult to fully utilize this promising approach. Current gaps in the literature on 7-ɑ-dehydroxylation include an incomplete understanding of the genes involved, the bacterial species that facilitate this biotransformation, and the pathway by which 7-ɑ-dehydroxylation is performed. Below we outline what is and is not known about the genes involved, followed by a review of the proposed pathways by which these gene products are thought to function in the processing of secondary bile acids.

## Bai operon

Ridlon et al. (2010) isolated and analyzed the bai operon derived from *C. hylemonae*. Though *C. hylemonae* is a bacterial species with low 7-ɑ-dehydroxylation activity, they reported high gene conservation with other high activity strains such as *C. scindens* (Ridlon et al., 2010). The isolated operon from *C. hylemonae* contains seven genes (*baiB*, *baiCD*, *baiE*, *baiF*, *baiG*, *baiH*, and *baiI*) and was instrumental in showing that 7-ɑ-dehydroxylating genes (Table 1) are inducible by primary bile acids. However, due to its high 7-ɑ-dehydroxylation activity, *C. scindens* is more commonly studied in gut bacterial bile acid metabolism; its bai operon, which we refer to as the canonical bai operon, also includes *baiA* and is labeled as baiBCDEAFGHI (Ridlon et al., 2010). In the following section we will describe what is known and what is debated about the function of each of these genes, going in the order in which they appear in the *C. scindens* genome. Notably, there are several proposed sequences in which these enzymes are thought to perform 7-ɑ-dehydroxylation and these divergent pathways will be described in the following section.

**Table 1:** Canonical bai operon genes and their encoded enzymes.

Gene	Enzyme	Proposed function
*Canonical Bai Operon*		
*baiB*	Bile acid CoA-ligase	Facilitates formation of a bile acid-CoA thioester
*baiCD*	NAD + -dependent 3-oxo-Δ^4^-cholenoic acid oxidoreductase	Catalyzes oxidation reaction to produce a 3-dehydro-Δ^4^-CA-CoA intermediate
*baiE*	Bile acid 7-α dehydratase	Performs diaxial trans elimination of water in 3-oxo-4-cholenoic acid substrates
*baiA*	3-α hydroxysteroid dehydrogenase	Catalyzes C3-hydroxyl group oxidation
*baiF*	Bile acid-CoA hydrolase; bile acid-CoA transferase	Acts on cholyl-CoA substrates to hydrolyze bile acid CoA conjugates
*baiG*	Bile acid membrane transport protein	Facilitates transport of bile acids across cell membrane
*baiH*	NADH:flavin oxidoreductase activity	Catalyzes production of a 3-dehydro-Δ4-CA-CoA intermediate
*baiI*	Bile acid 7-β dehydratase; Δ^5^-ketosteroid isomerase	–

### baiB

*baiB* is the first open reading frame (ORF) in the canonical bai operon of *Clostridium scindens*. Encoding bile acid CoA-ligase, *baiB* is thought to catalyze the first step in the 7-α-dehydroxylation pathway by facilitating the formation of a bile acid-CoA thioester (Mallonee et al., 1992). ATP is required for catalysis, and both AMP and pyrophosphate are additional reaction products. While bile acid CoA-ligase works on CA, DCA, and CDCA, data suggests that this enzyme functions with a variety of different bile acids that have accessible C-24 carboxyl substituents (Mallonee et al., 1992). The *baiB* gene shares sequence similarity with several other polypeptides, including *entE* which encodes 2,3-dihydroxybenzoate-AMP ligase in *E. coli* and *grsA* which encodes Gramicidin S synthetase 1 in *B. brevis*, among others (Mallonee et al., 1992). Further work is required to determine the function of bacterial genes with sequence similarity to *baiB*.

### baiCD

*baiCD* is located directly downstream of *baiB* in the canonical bai operon. A prior study by Kang et al. (2008) provided evidence to suggest that the *baiCD* gene product facilitates the production and action of NAD + -dependent 3-oxo-Δ^4^-cholenoic acid oxidoreductases that recognize 7-ɑ-hydroxy bile acids. Though competing hypotheses exist, several studies support that *baiCD* encodes an enzyme that catalyzes C4-C5 oxidation to produce a 3-dehydro-Δ^4^-CA-CoA intermediate. Kang and colleagues also produced key findings that revealed a stereochemical component of substrate recognition for 7-ɑ and 7-β hydroxy groups, as 7-ɑ hydroxy groups are positioned axially and 7-β hydroxy groups are positioned equatorially. Kang et al. (2008) proposed that the stereochemical differences found among *C. scindens* and *C. hiranonis* species provides evidence to suggest that the oxidative portion of the 7-ɑ-dehydroxylation pathway contains two substrate-dependent branches. A recent review paper by Guzior and Quinn (2021) agreed upon the role of *baiCD* in C4 oxidation within the 7-ɑ-dehydroxylation pathway, with *baiH* carrying out a homologous function during 7-β-dehydroxylation, which involves the removal of the 7β-hydroxy group of ursodeoxycholic acid, a 7β-epimer of CDCA (White et al., 1982).

### baiE

Located directly downstream of *baiCD*, *baiE* encodes a bile acid 7-ɑ dehydratase (Wells and Hylemon, 2000). By expressing *baiE* derived from the *C. scindens* genome in *E. coli*, Dawson et al. (1996) were able to identify its encoded enzyme, a 19.5 kDa 7-ɑ dehydratase that primarily acts on 7ɑ, 12ɑ-dehydroxy-3-oxo-4-cholenoic acid and 7-ɑ-hydroxy-3-oxo-4-cholenoic acid substrates. While the exact enzymatic mechanisms had not been previously investigated at the time of this study, the proposed mechanism involved the diaxial trans elimination of water as the fourth step in the 7-ɑ-dehydroxylation pathway. Ridlon et al. (2006) also suggested that the enzyme encoded by *baiE* is stereospecific and contains a homologous amino acid sequence to the *baiI* gene product, a proposed 7-β dehydratase with similar function.

### baiA/baiA2

Continuing downstream in the canonical bai operon, *baiA2* encodes a 3-ɑ-hydroxysteroid dehydrogenase that catalyzes C3-hydroxyl group oxidation (Bhowmik et al., 2014). A study by Bhowmik et al. (2014) revealed a 92% sequence similarity to the gene product of *baiA1*, another 3-ɑ hydroxysteroid dehydrogenase; both genes are included in the short chain dehydrogenase/reductase (SDR) class of proteins that use NAD or NADP as a co-factor for enzymatic activity. Their findings also revealed that *baiA2* exclusively uses NAD as a co-factor due to the structural makeup of the co-factor binding site. Bhowmik et al. (2014) additionally showed that *baiA2* preferentially catalyzes the reduction reaction. However, as enzymatic activity showed pH-dependence, it was hypothesized that *baiA2* may also catalyze the oxidative reaction of the pathway. A study by Ridlon and Hylemon (2012) also identified multiple copies of the *baiA* gene within the *C. scindens* VPI 12708 chromosome, including a *baiA3* gene, and agreed upon suggestions that *baiA* genes maintain a strong preference for bile acid-CoA conjugate substrates when compared to non-CoA conjugates.

### baiF

Downstream of *baiA2*, *baiF* encodes a bile acid-CoA hydrolase that acts on cholyl-CoA, 3-dehydrocholyl-CoA, and chenodeoxycholyl-CoA substrates (Ye et al., 1999). The encoded bile acid-CoA hydrolase shares significant amino acid similarity with carnitine dehydratase in *E. coli*, though early studies did not find motifs characteristic of thioesterases in the amino acid sequence of the enzyme encoded by *baiF*; this provided evidence to suggest that *baiF* may encode a novel group of thioesterases (Ye et al., 1999). Later studies by Ridlon and Hylemon alternatively suggest that the *baiF* gene product acts as bile acid CoA transferase (Ridlon and Hylemon, 2012). Using high performance liquid chromatography, Ridlon and Hylemon provided strong evidence that the gene product of *baiF* transfers CoA from a deoxycholic acid-CoA conjugate to *CA*. They also revealed that the encoded transferase can act on a broad variety of bile acid substrates though is most active when the bile acid CoA donor is a homologous secondary bile acid, such deoxcholyl-CoA.

### baiG

Next in the bai operon, *baiG* encodes a protein that imports bile acids across the cell membrane for use in 7-α-dehydroxylation and that contains sequence homology to membrane transport proteins, specifically the transmembrane facilitator superfamily (Mallonee and Hylemon, 1996). Interestingly, *baiG* reported the highest sequence homology with antibiotic resistance proteins within this superfamily (Mojumdar and Khan, 1988). *baiG* has been shown to import CA, CDCA, DCA, cholyglycine and 7-oxo-cholic acid (Mallonee and Hylemon, 1996). When cloned in *E. coli*, *baiG* exhibited greater efficiency for import of CA and CDCA compared to that for DCA, cholylglycine, and 7-oxo-cholic acid. Though not well understood, studies suggest that *baiG* may also be involved in bile acid export, as the absence of *baiG* has been associated with accumulation of cholic acid within bacterial cells (Thanassi et al., 1997). However, a previous study showed that *baiG* independent bile acid export is also possible (Mallonee and Hylemon, 1996).

Though the mechanistic processes carried out by the aforementioned bai genes, including *baiB*, *baiCD*, *baiE*, *baiA/baiA2*, *baiF*, and *baiG*, remain incompletely understood, their functions have generally been well-established over years of investigation by numerous research groups. As we continue forward in this review, it is important to note that the following genes in the canonical bai operon, as well as the more recently discovered baiJKL operon, are not as established in the literature and more commonly involve indeterminate viewpoints regarding function and mechanism.

### baiH

Downstream of *baiG*, *baiH* encodes a protein that carries out NADH:flavin oxidoreductase activity (Franklund et al., 1993). The *baiH* gene contains sequence homology to NADH oxidase from *T. brockii* and, interestingly, strong sequence similarity exists between the *baiH* and *baiCD* genes as well as their gene products (Franklund et al., 1993). Furthermore, when the purified *baiH* gene product was introduced to *C. scindens*, Franklund et al. (1993) reported decreases in the ratio of oxidized to reduced 7-α-dehydroxylation bile acid intermediates. This study provided evidence to suggest that the enzyme encoded by *baiH* plays a role in cellular NAD+/NADH regulation. In a later study by Kang et al., cloning of *baiH* from the *C. scindens* genome in *E. coli* provided evidence that the *baiH* gene product catalyzes the oxidation of 7-β-hydroxy-3-oxochol-24-oyl-CoA in a stereospecific manner (Kang et al., 2008). As a whole, this study supports a role for *baiH* in the oxidation of C4-C5 during 7-α-dehydroxylation, thereby catalyzing the production of a 3-dehydro-Δ^4^-CA-CoA intermediate. This finding differed from those of prior studies suggesting that *baiH* facilitates the reduction of a C-C double bond (Kengen et al., 2003).

### baiI

The most downstream gene within the bai operon, *baiI* has recently been hypothesized to encode a Δ (Eisenstein, 2020)-ketosteroid isomerase; however, a prior study by Kang et al. (2008) predicted that the *baiI* gene product was a bile acid 7-β dehydratase due to sequence homology with the *baiE* gene when studied in *C. scindens*. Interestingly, Funabashi et al. (2020) also recently reported findings suggesting that *baiI* is not required for 7-α-dehydroxylation of CA *in vitro* despite its strong conservation in species known to perform dehydroxylation.

### *baiJKL* operon

Ridlon and Hylemon (2012) reported the discovery of a second multigene operon involving a bile acid CoA transferase. By sequencing upstream of the second copy of the *baiA* gene, an individual gene induced by cholic acid, they identified an additional set of genes potentially involved in bile acid metabolism. These reports initially applied to *C. hylemonae*; however, this set of genes was also identified in *C. scindens* (VPI 12708) and shared 89% amino acid sequence with those found in *C. hylemonae*.

*baiL* is the ORF located first upstream of the *baiA* gene and is thought to encode a short chain pyridine nucleotide-dependent alcohol/polyol oxidoreductase; this family of oxidoreductases includes that encoded by the *baiA* gene (Ridlon and Hylemon, 2012). Positioned upstream of *baiL*, *baiK* is thought to encode a CoA transferase (type III) due to its significant shared sequence identity with the protein encoded by *baiF* and other type III CoA transferases (Ridlon and Hylemon, 2012). Upstream of *baiK*, *baiJ* is believed to encode a flavoprotein resembling a 3-ketosteroid-Δ (Goyal and Jialal, 2022)-dehydrogenase (Ridlon and Hylemon, 2012). Notably, Ridlon and Hylemon suggested that these genes may not be present in some *C. scindens* strains, as genome sequencing of *C. scindens* ATCC 35704 did not detect baiJKL genes despite performing 7-ɑ-dehydroxylation (Ridlon and Hylemon, 2012). This finding calls into question the necessity of the baiJKL operon in this metabolic process. Evidence related to *baiJ* has been limited, however, a recent study by Ridlon identified a *baiJ* homolog in *C. hiranonis* thought to encode a flavin-dependent oxidoreductase. Nevertheless, further investigation is needed to gain a better understanding of this gene and its corresponding gene product (Ridlon et al., 2020).

### baiN

Most prior studies have focused on the aforementioned canonical bai operon, though these genes do not provide data on the reductive reaction pathway in 7-ɑ-dehydroxylation. A study by Harris et al. (2018) recently reported the identification of a *baiN* gene thought to encode a recombinant flavoprotein in *C. scindens* ATCC 35704. Their investigation revealed a likely involvement of *baiN* in the reductive arm of 7-ɑ-dehydroxylation, facilitating the reduction and metabolism of C6-C7 and C4-C5 bonds in the A and B rings of bile acid. *baiN* was also identified in *C. hylemonae*, as well as 7-ɑ dehydroxylating gut bacteria by phylogenetic studies.

## 7-α-dehydroxylation pathways

**Figure 1:**
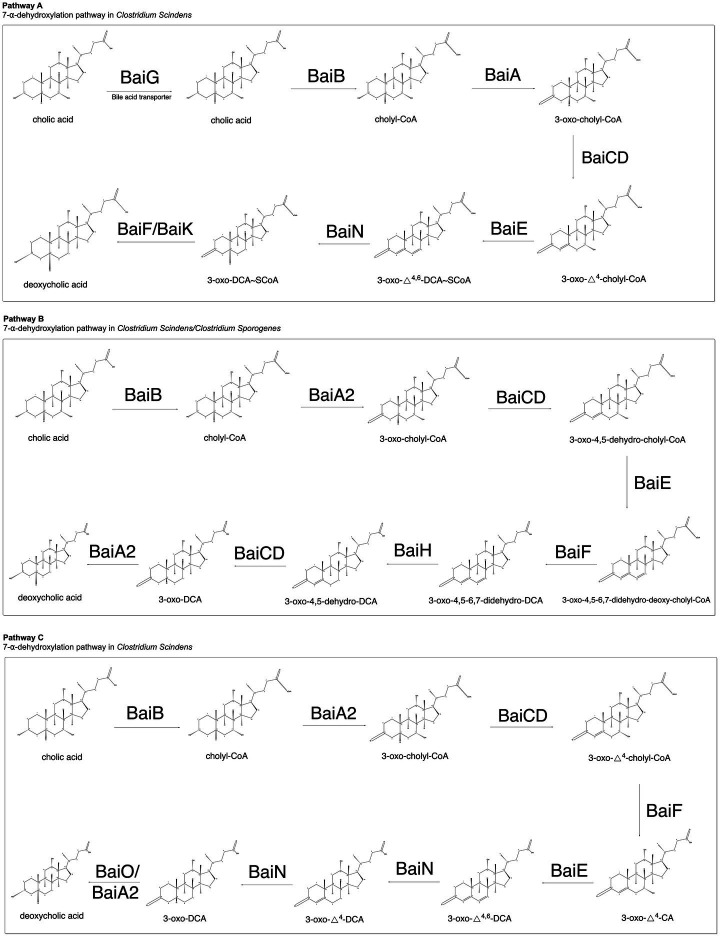
Summary of proposed 7-α-dehydroxylation pathways. Studies presented in each pathway are cited accordingly. Pathway A (Ridlon et al., 2006, 2010, 2014, 2020; Ridlon and Hylemon, 2012), Pathway B (Funabashi et al., 2020), and Pathway C (Guzior and Quinn, 2021).

After analyzing the proposed 7-α-dehydroxylation pathways outlined among the landmark bile acid metabolism papers currently available in the literature, we have identified several steps in 7-α-dehydroxylation that demonstrate consensus among the proposed pathways, as well as several that suggest divergent reaction mechanisms worthy of further investigation. Each step is summarized in Figure 1 and is outlined in further detail below.

### Bile acid import

#### Step 0

There is strong agreement among currently proposed 7-α-dehydroxylation pathways that the initial component of gut bacterial bile acid metabolism involves a H^+^-dependent bile acid transporter encoded by *baiG*. Prior investigations have reported that bacteria cloned with the *baiG* gene show increased uptake of cholic and chenodeoxycholic acid, while taking up low levels of deoxycholic acid and 7-oxocholic acid (Mallonee and Hylemon, 1996). *baiG* is thus theorized to facilitate the transport of unconjugated bile acids into the interior of the bacterial cell for 7-α-dehydroxylation, while a different transporter may facilitate transport of bile acids out of the cell.

## Oxidative arm

### Step 1

Following transport of unconjugated primary bile acids into the interior of the cell membrane, cholic acid is thought to be transformed into cholyl-CoA by *baiB*-facilitated ATP-dependent CoA ligation. Prior studies by Mallonee et al. (1992) suggest that the encoded bile acid-CoA ligase catalyzes the first step of 7-α-dehydroxylation by utilizing ATP to activate and produce a bile acid-CoA thioester, as well as AMP and pyrophosphate by-products. Cholic acid ligation has also been observed with the use of ADP, though reaction rates were slower when compared to ligation with ATP input (Mallonee et al., 1992). Interestingly, a model for the 7-α-dehydroxylation pathway in *C. scindens* VPI 12708 proposed by Ridlon and Hylemon (2012) also suggested a potential role of *baiF*/*baiK* in catalyzing the formation of cholyl-CoA from *CA*. While *baiF* and *baiK* activity were observed after cloning and expression in *E. coli* rather than *C. scindens*, Ridlon and Hylemon found that both *baiF* and *baiK* encoded bile acid CoA transferases with fairly widespread substrate specificity (*baiK* being more specific than *baiF*). As CoA transferases are capable of conserving thioester bond energy and thus reducing the number of ATP molecules required for reaction catalysis, Ridlon and Hylemon theorized that ATP-independent transfer of CoA to cholic acid may rapidly replace ATP-dependent CoA ligation to produce the product of step 1 in the 7-ɑ-dehydroxylation pathway (Ridlon and Hylemon, 2012).

### Step 2

The second step of 7-α-dehydroxylation is thought to involve the oxidation of a C3-hydroxyl group to produce a C3-oxo bile acid intermediate. Encoded by *baiA*, a 3-α hydroxysteroid dehydrogenase uses NAD as a preferred co-factor due to binding site conformation (Sun et al., 2018). In agreement with Mallonee and colleagues’ findings, Bhowmik and colleagues found that *baiA1* and *baiA2* prefer bile acid-CoA ester substrates (Bhowmik et al., 2014). The observation that the encoded *baiA* enzyme uses substrates containing CoA in combination with the hypothesis that 7-ɑ-dehydroxylation is initiated by CoA ligation to CA provided strong evidence for this step in the pathway. While most prior studies did not identify the specific *baiA* gene responsible for catalyzing this reaction step (*baiA1*, *baiA2*, and/or *baiA3*), the 7-ɑ dehydroxylation pathway proposed by Funabashi et al. (2020) specifically identified *baiA2* as a gene that can facilitate completion of this step of the pathway.

### Step 3

A NAD(H)-dependent 7-ɑ-hydroxy-3-oxo-Δ^4^-cholenoic acid oxidoreductase is then encoded by *baiCD* and is stereospecific for 7-ɑ-hydroxy bile acids. Though further investigation is needed to better identify the specific oxidoreductase involved in this process, studies have shown that the resulting enzymatic reaction produces 3-oxo-Δ^4^-cholenoic acid-CoA intermediates *via* a second oxidation of Cholyl-CoA (first oxidation step occurs in step two by *baiA*) (Kang et al., 2008).

### Step 4

Following production of a 3-oxo-Δ-4-cholenoic acid-CoA intermediate, *baiE* encodes bile acid 7-ɑ dehydratase. A study by Dawson et al. (1996) identified and characterized this enzyme, suggesting that it facilitates the fourth step of 7-ɑ-dehydroxylation by dehydrating the intermediate produced in step three and thus producing a 3-oxo-Δ^4,6^ bile acid CoA intermediate. The *baiE* encoded enzyme is the rate-determining enzyme in the 7-ɑ-dehydroxylation pathway (Bhowmik et al., 2016). Further, the removal of the 7-ɑ hydroxy group from the intermediate produced by *baiCD* through release of water completes the oxidative arm of 7-ɑ-dehydroxylation (Bhowmik et al., 2016).

## Reductive arm

Following completion of the oxidation arm of the pathway, 7-ɑ-dehydroxylation involves a series of reductive steps to produce secondary bile acid end products. While most papers reporting on this metabolic process propose pathways that largely agree in steps zero through four, there is greater divergence among the proposed reductive steps in the 7-ɑ-dehydroxylation pathway. Doden et al. (2018) proposed a series of reductive steps catalyzed by a *baiN* encoded flavoprotein identified by Harris et al. (2018) in *C. scindens* ATCC 35704 earlier that year. Harris et al. (2018) produced findings consistent with the involvement of *baiN* in the reductive steps following 7-ɑ dehydration, showing that the encoded flavoprotein is involved in the reduction of two double-bonds of the bile acid A and B rings (C6-C7 and C4-C5). Their preliminary findings predicted that the encoded flavoprotein is a flavin-dependent squalene desaturase, which facilitates the formation of 3-dehydro-deoxycholic acid *via* two reduction steps. Though Harris et al. (2018) indicates a final step to transform 3-dehydro-DCA to DCA, the enzyme facilitating this transformation had not been identified at the time of publication.

In contrast to the pathway proposed by Doden et al. (2018), Harris et al. (2018), and Funabashi et al. (2020) demonstrated that a different sequence of the bai genes is sufficient for the performance of 7-ɑ-dehydroxylation that involves several additional steps following *baiE* activity and resulting completion of the oxidative arm of the pathway. An important point to note, however, is that Funabashi et al. (2020) investigated 7-ɑ-dehydroxylation by cloning the bai operon from *C. scindens* and genetically introducing it into *C. sporogenes.* This approach is different from approaches used in prior studies as the incorporation of a genetically engineered bai operon into a new bacterial species does not involve uptake of other endogenous factors that may influence the 7-ɑ-dehydroxylation pathway. Funabashi et al. (2020) suggest that the reduction in 7-ɑ-dehydroxylation efficiency seen with the cloned bai operon is attributable to the absence of other important endogenous factors that remain unknown. In contrast to prior proposed pathways, their findings suggest that after the last oxidation step by *baiE*, *baiF* encodes a CoA transferase that transforms 3-oxo-4,5-6,7-didehydro-deoxy-cholyl CoA to 3-oxo-4,5-6,7-didehydro-DCA. Following enzymatic activity by *baiF*, *baiH* is theorized to encode a Fe-S flavoenzyme that catalyzes the first reduction in a two-step reductive process to produce 3-oxo-4,5-dehydro-DCA. Their data provided evidence to suggest that an additional enzyme is required to complete the two-step reductive process, with *baiCD* being a likely candidate as it is homologous to *baiH*. Addition of *baiCD* resulted in further reduction to 3-oxo-DCA and was thus proposed as a reduction step in the 7-ɑ-dehydroxylation pathway. The last step in the pathway as proposed by Funabashi et al. (2020) involves the reduction of 3-oxo-DCA to DCA by a *baiA2* encoded hydroxysteroid dehydrogenase. Interestingly, as opposed to other suggested 7-ɑ-dehydroxylation pathways, this pathway proposes that *baiA2* and *baiCD* each function twice in the 7-ɑ-dehydroxylation pathway and have important roles in both the oxidative and reductive arms. While their work demonstrates that this sequence of steps is sufficient to convert CA to DCA, the *in vivo* efficiency of this pathway is lower than that observed in *C. scindens*.

Most recently, Guzior and Quinn published a review in 2021 on human bile acid transformations by the microbiome with an alternative pathway proposed for 7-ɑ-dehydroxylation in *C. scindens* (Guzior and Quinn, 2021). Though identical to the aforementioned pathways in Steps 1–3 (oxidative arm), Guzior and Quinn suggest that *baiF* acts prior to *baiE*, while both of the pathways proposed by Doden and Funabashi include *baiE* acting before *baiF*. Accordingly, in Guzior and Quinn’s model, *baiF* encodes a CoA transferase that catalyzes the formation of 3-oxo-Δ^4^-CA and cholyl-CoA from 3-oxo-Δ^4^-cholyl-CoA (Doden et al., 2018; Funabashi et al., 2020; Guzior and Quinn, 2021). Following activity by *baiF*, *baiE* encodes a 7-ɑ dehydratase that facilitates the rate-limiting step of dehydroxylation of C7, similar to alternatively proposed pathways. Following completion of the oxidative arm of the pathway, Guzior and Quinn propose that *baiN* catalyzes two successive reductive steps to transform the intermediate produced by dehydroxylation into 3-oxo-Δ^4^-DCA (step 1) and 3-oxo-DCA (step 2) (Guzior and Quinn, 2021). Like the pathway proposed by Funabashi, Guzior and Quinn propose that *baiA2* is involved in the final redox reaction that transforms 3-oxo DCA into DCA. However, though not yet experimentally validated, Guzior and Quinn reference a recent study by Heinken et al. (2019) that provided evidence to suggest that *baiO* may function similarly to *baiA2* by completing the last reduction in the 7-ɑ-dehydroxylation pathway *via* activity by an encoded NADH-dependent reductase.

### LCA production

While 7-α-dehydroxylation refers to the production of both DCA and LCA, the majority of work on 7-ɑ-dehydroxylation has focused on DCA production. Nevertheless, research suggests that there are important differences in the production of LCA versus DCA. While there are gut bacterial species and 7-α-dehydroxylation enzymes that can use both CA and CDCA as a substrate in the generation of secondary bile acids, there are gut bacterial species and 7-α-dehydroxylation gene products that can only react with CA (Hirano et al., 1981; Ridlon and Hylemon, 2012). Though further research is needed to better elucidate differences in bile acid-specific 7-α-dehydroxylation pathways, a study by Ridlon and Hylemon (2012) showed that a recombinant *baiK* gene selectively used a DCA-CoA substrate, demonstrating a point of divergence dependent on the processing of CA or CDCA. Additionally, Hirano et al. (1981) previously reported differential dehydroxylation activity of CA and CDCA in *Clostridium*, though the bacterial strain was unidentified at the time. Interestingly, a recent study by Sato et al. (2021) investigating bile acid metabolism in centenarians identified a route for transformation of 3-oxoallo-LCA to allo-LCA and isoallo-LCA *via* activity by a 5-α reductase (5AR) homolog and a 3-α/3-β hydroxysteroid dehydrogenase (3β-HSDH-I), respectively. Their findings showed that in isolated bacterial strains with gene clusters related to bile acid metabolism, clusters with 5αR and 3β-HSDH-I were associated with *in vitro* production of 3-oxoalloLCA and isoalloLCA from 3-oxo-Δ^4^-LCA. Similar to patterns seen in DCA production, production efficiency of LCA was largely dependent on bacterial strain and substrate specificity.

## Summary

Currently available literature on gut bacterial bile acid metabolism and 7-ɑ-dehydroxylation represents decades of investigation and discoveries that have allowed for the creation of several different metabolic pathways to mechanistically explain the transformation of primary to secondary bile acids by the gut microbiome. While close analyses of the literature to date reveals many consistencies in both enzymes involved and the intermediates that are sequentially produced, there remain several gaps in knowledge that necessitate further investigation in order to effectively target this pathway for therapeutic intervention. As more recently proposed pathways often include newly discovered bai genes and/or additional enzymes involved in this process, further study of the potential genes involved in 7-ɑ-dehydroxylation outside of the canonical bai operon may provide valuable information on both gut bacterial bile acid metabolism and its role in the context of other metabolic pathways and functions. Additionally, as different bacterial species are known to demonstrate varying levels of 7-ɑ-dehydroxylation activity, further investigation of genes outside of the bai operon may aid in better understanding the factors that influence metabolic efficiency beyond a simple dichotomy that characterizes particular species as either being able to perform 7-ɑ-dehydroxylation or not being able to. Further experimental manipulation of bai genes may also elucidate gene-specific contributions to 7-ɑ-dehydroxylation activity, bile acid pool composition, and metabolic regulation. Moreover, whether and how different bacterial strains may cooperate with one another in bile acid metabolism is poorly understood. As such, the dynamic regulation of this pathway in response to environmental and host factors such as diet and disease state requires further investigation. Despite current gaps in knowledge, gut bacterial bile acid metabolism presents a promising potential avenue for the discovery and development of novel therapeutic targets for metabolic disease.

## Author contributions

JW and BC conceived the manuscript, reviewed and edited the manuscript. JW prepared the original draft. BC finalized the manuscript. All authors contributed to the article and approved the submitted version.

## Funding

This work was supported by the National Center for Complementary and Integrative Health of the National Institutes of Health under Award number R21AT010956. The content is solely the responsibility of the authors and does not necessarily represent the official views of the National Institutes of Health.

## Conflict of interest

The authors declare that the research was conducted in the absence of any commercial or financial relationships that could be construed as a potential conflict of interest.

## Publisher’s note

All claims expressed in this article are solely those of the authors and do not necessarily represent those of their affiliated organizations, or those of the publisher, the editors and the reviewers. Any product that may be evaluated in this article, or claim that may be made by its manufacturer, is not guaranteed or endorsed by the publisher.
